# Modeling the Cost of Vaccinating a Measles Zero-Dose Child in Zambia Using Three Vaccination Strategies

**DOI:** 10.4269/ajtmh.23-0412

**Published:** 2024-05-21

**Authors:** Joshua Mak, Bryan N. Patenaude, Simon Mutembo, Monica E. Pilewskie, Amy K. Winter, William J. Moss, Andrea C. Carcelen

**Affiliations:** ^1^ International Vaccine Access Center, Johns Hopkins Bloomberg School of Public Health, Baltimore, Maryland;; ^2^ Department of International Health, Johns Hopkins Bloomberg School of Public Health, Baltimore, Maryland;; ^3^ Center for Indigenous Health, Johns Hopkins Bloomberg School of Public Health, Baltimore, Maryland;; ^4^ Department of Epidemiology & Biostatistics, University of Georgia College of Public Health, Athens, Georgia;; ^5^ Department of Epidemiology, Johns Hopkins Bloomberg School of Public Health, Baltimore, Maryland

## Abstract

Countries with moderate to high measles-containing vaccine coverage face challenges in reaching the remaining measles zero-dose children. There is growing interest in targeted vaccination activities to reach these children. We developed a framework for prioritizing districts for targeted measles and rubella supplementary immunization activities (*SIA*s) for Zambia in 2020, incorporating the use of the WHO’s Measles Risk Assessment Tool (*MRAT*) and serosurveys. This framework was used to build a model comparing the cost of vaccinating one zero-dose child under three vaccination scenarios: standard nationwide SIA, targeted subnational SIA informed by MRAT, and targeted subnational SIA informed by both MRAT and measles seroprevalence data. In the last scenario, measles seroprevalence data are acquired via either a community-based serosurvey, residual blood samples from health facilities, or community-based IgG point-of-contact rapid diagnostic testing. The deterministic model found that the standard nationwide SIA is the least cost-efficient strategy at 13.75 USD per zero-dose child vaccinated. Targeted SIA informed by MRAT was the most cost-efficient at 7.63 USD per zero-dose child, assuming that routine immunization is just as effective as subnational SIA in reaching zero-dose children. Under similar conditions, a targeted subnational SIA informed by both MRAT and seroprevalence data resulted in 8.17–8.35 USD per zero-dose child vaccinated, suggesting that use of seroprevalence to inform SIA planning may not be as cost prohibitive as previously thought. Further refinement to the decision framework incorporating additional data may yield strategies to better target the zero-dose population in a financially feasible manner.

## INTRODUCTION

In situations where nonselective mass measles and rubella vaccination campaigns yield small advances in vaccination coverage, targeted immunization strategies may be more efficient for outbreak prevention. “Zero-dose” children, commonly defined as either those who were never vaccinated with the first dose of the diphtheria, tetanus, and pertussis vaccine or those who were never vaccinated with a measles vaccine dose,^1^ are particularly at risk of infection and contributing to ongoing transmission. Nationwide, nonselective supplementary immunization activities (SIAs; or mass vaccination campaigns) are unlikely to most efficiently reach zero-dose children, as many factors inhibiting access to routine immunization (RI) services may also hinder access during vaccination campaigns.^2^ This issue has generated interest in tailored SIAs, which seek to increase vaccination coverage in a specific subset of the population (e.g., geographic location or age group) through targeted approaches.

Zambia is well suited for implementation and evaluation of targeted SIAs. Despite periodic nationwide, nonselective measles and rubella SIAs and high national coverage with the first dose of measles-containing vaccine (MCV), children and communities with low MCV coverage hinder measles elimination.^3^ Targeted vaccination strategies could more efficiently and effectively identify and vaccinate measles zero-dose children and missed communities with high measles outbreak risk. Ideally, such areas would be prioritized based on immunity profiles, although these are difficult to infer from routinely available data.^4^ Some children receiving MCV1 may not develop protective immunity, and unvaccinated children who survive measles acquire immunity upon recovery.^5^ Administrative or survey vaccination coverage data, often acquired via immunization card or caregiver recall, is frequently of poor quality.^2^^,^^6^

Serosurveys provide direct estimates of measles immunity.^5^^–^^7^ Measles serosurveys may help guide decision-makers in prioritizing districts with low measles seroprevalence for targeted SIAs while allowing adequately performing areas to improve their RI systems with fewer financial and human resource investments than required to conduct nationwide SIAs. Although there is little published literature exploring the use of measles seroprevalence data to guide targeted SIAs, evidence with dengue vaccine suggests it is a viable option.^8^ Yet, widespread adoption of serological surveys is hindered by time and resource limitations for planning and implementation. In practice, the feasibility of acquiring seroprevalence data may be improved by nesting serosurveys within other nationally representative surveys, such as demographic health surveys (DHSs) or other programmatic surveys,^4^^,^^9^^,^^10^ or through the use of residual specimens collected for other purposes.^11^^–^^14^

The cost-effectiveness literature on measles and rubella SIAs in low- and middle-income countries (LMICs) is scarce, component costs for vaccination programs are poorly understood, and “cost-effective” interventions are context dependent.^15^^–^^17^ The WHO recommends that countries with high outbreak risk should conduct nationwide, nonselective SIAs.^16^^,^^18^ However, nonselective SIAs may be less effective in countries with high RI coverage, and the choice between using nationwide or targeted subnational SIAs is complex. Therefore, it is essential to optimally triangulate surveillance, vaccination, and local epidemiologic data to guide targeted measles vaccination efforts,^18^ and seroprevalence data may be a valuable additional data source.

We present a basic framework for prioritizing districts in Zambia for targeted subnational measles and rubella SIAs, including the potential use of measles seroprevalence estimates. We also conducted a cost comparative analysis to determine the total cost of a vaccination campaign and extrapolate the cost of vaccinating one measles zero-dose child under several vaccination scenarios, including a nationwide mass vaccination campaign, targeted district-level risk assessment, and focal measles serosurveys.

## MATERIALS AND METHODS

Our model estimated the cost of targeted district-level SIAs to reach measles zero-dose children relative to the use of a nationwide, nonselective measles SIA in Zambia over 1 month in June of 2020, assuming no disruptions to vaccination owing to the COVID-19 pandemic. The cost per measles zero-dose child vaccinated is a function of the total cost of an SIA intervention (reaching children who were previously vaccinated against measles and children who were measles zero-dose) divided by the total number of measles zero-dose children reached by the SIA. This model targets children younger than 5 years at the district level. Economies of scale are not considered where costs are concerned. This model was run using R v. 4.2.1 with RStudio v. 2021.9.2. For district-level descriptive statistics, please see Supplement A.

### Vaccination scenarios.

**Figure 1.**
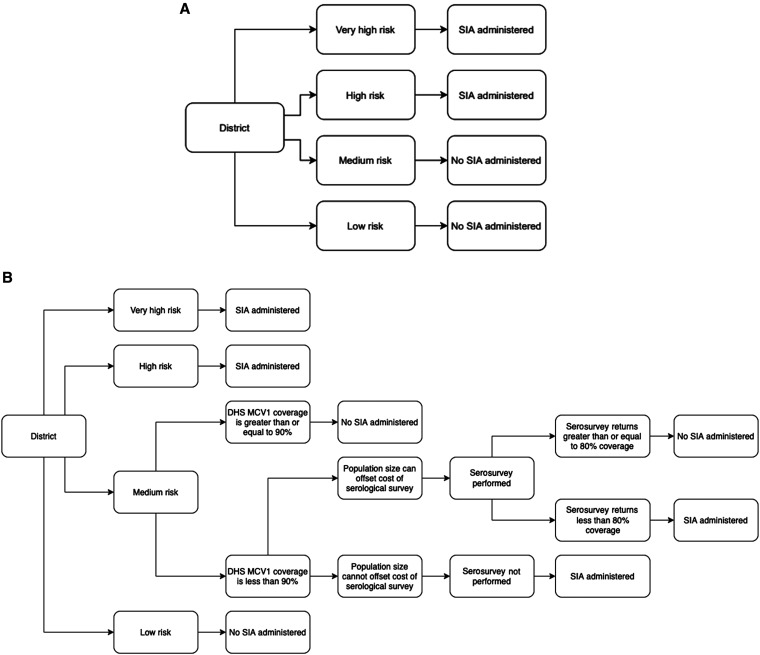
(**A**) Scenario 2 decision tree. All Zambian districts classified by MRAT as having “high risk” or “very high risk” of measles outbreak in 2020 conducted an SIA. All other districts incurred no SIA costs. (**B**) Scenario 3 decision tree. Districts classified by the WHO’s MRAT as “low risk” continued routine immunization, and high-risk and very high–risk districts conduct an SIA. “Medium-risk” districts with low DHS MCV1 coverage rates assessed for whether the cost of implementing a selective SIA would be lower than that of conducting a measles serosurvey. Among medium-risk districts chosen for a measles serosurvey, the measles seroprevalence estimate determined whether an SIA should be administered or not. DHS = demographic health survey; MCV = measles-containing vaccine; MRAT = Measles Risk Assessment Tool; SIA = supplementary immunization activity.

We considered three measles vaccination scenario strategies. Scenario 1 (S1) was a nationwide, nonselective SIA, the standard mass vaccination campaign used in LMIC settings. Scenario 2 (S2) used the WHO’s Measles Risk Assessment Tool (MRAT) to classify Zambian districts as low, medium, high, and very high risk for a measles outbreak (Figure 1A).^19^ In S2, only high-risk and very high–risk districts were marked for district-level SIAs, whereas low- and medium-risk districts focused on immunizing zero-dose children via RI systems. Note that districts not selected for subnational SIA continued with RI, which administered vaccines only to children who were zero-dose. The proportion of measles zero-dose children among the total population of children vaccinated through an SIA was dependent on each district’s measles outbreak risk classification. Because enumeration of the measles zero-dose population is difficult, the proportion of children reached via subnational SIA who were zero-dose was assumed to be tiered, with very high–outbreak risk districts having the greatest proportion of measles zero-dose children.

Scenario 3 (S3) built upon S2 to incorporate the use of measles serosurveys (Figure 1B). High- and very high–risk districts were selected for an SIA, and low-risk districts continued RI. Medium-risk districts were ranked using DHS MCV1 coverage estimates.^20^ Medium-risk districts with MCV1 coverage less than 90% were selected for a serosurvey, except for those with a maximum threshold population size that did not justify the use of a serological survey: 9,594 for a community-based serological survey, 4,345 for a residual-based serological survey, and 8,379 for an IgG point-of-contact diagnostic test. Districts with small population sizes, such that it would cost less to implement a subnational SIA than to administer a serosurvey, received a district-level SIA. For derivation of these population sizes, see Supplement B.

The remaining districts implemented a measles serosurvey. Among these, districts with measles seroprevalence less than 80% receive a subnational SIA, whereas those with a seroprevalence greater than 80% continued RI. As in S2, the proportion of measles zero-dose children reached as a proportion of the total vaccinated population in the SIA depended on the district’s outbreak risk classification. For S3, three serology sampling scenarios were considered: S3a simulated community-based serological surveys, S3b simulated the use of residual blood specimens from health facilities, and S3c simulated the use of measles IgG point-of-contact diagnostic tests.

### Measles risk assessment tool.

Our decision analysis relied upon the WHO’s MRAT to classify the outbreak risk of each district in Zambia.^19^ This Excel-based platform draws upon both administrative data and characteristics that can contribute to a measles outbreak to assign each district a measles outbreak risk score. Score thresholds classified each district as being at low, medium, high, or very high risk of a measles outbreak in 2020. For S3, we used 2018 Zambia DHS MCV1 coverage estimates to independently rank MRAT medium-risk districts by risk priority.

### Model input parameters and population characteristics.

District-level population demographic data were provided by Zambia’s Ministry of Health. The most recent district total population estimates were for 2018, although data were not available for all districts. Using the 2018 population estimate for each district, we applied a 3% annual growth rate to adjust for total district population in 2020.^21^ On average, 17.5% of Zambia’s overall population was younger than 5 years, and we assumed that this percentage applied equivalently for all 110 districts because age distribution data were only available for some districts. As a result of demographic data limitations, we assumed that the SIA targeted all children younger than 5 years.

To determine what percentage of the target population was reached by our simulated SIA, we applied provincial-level results of Zambia’s 2016 measles SIA to all districts. Among vaccinated children, a proportion were expected to be measles zero-dose prior to the SIA. Vaccination coverage data collected during the 2016 Zambia measles SIA using both immunization card and caregiver recall showed that 4% of children vaccinated during the measles and rubella SIA were zero-dose in rural settings and 8% in urban settings.^22^ When using only data from the immunization card, 13% of children in rural settings and 15% in urban settings were measles zero-dose children. Therefore, we assumed that the proportion of measles zero-dose children vaccinated as a proportion of all children vaccinated in the SIA was 4% for low-risk districts, 8% for medium-risk districts, 13% for high-risk districts, and 15% for very high–risk districts. Furthermore, we assumed that SIAs implemented in fewer districts would reach a greater proportion of children, regardless of zero-dose status, than traditional nationwide SIAs. This was accounted for by increasing the number of children vaccinated in S2 and S3 by 10% and 5%, respectively. For districts that were not selected for SIA and instead focused on investing in RI, the deterministic model assumed that an improved RI system reached as many zero-dose children as a subnational SIA, without the redundancies of vaccinating children who had previously received a dose of MCV. Basic district-level statistics used in the models are shown in Supplemental Table 1.

Total vaccination costs have two components: materials costs and delivery costs. Measles-containing vaccine material costs for measles-only vaccine were taken from Portnoy et al.^23^ These costs accounted for the price per dose of the vaccine, injection equipment, safety boxes, and freight charges. For Gavi-eligible countries, including Zambia, total material costs ranged from 0.28 to 0.35 USD (2020 adjusted) per child vaccinated.^23^ Our delivery cost estimates for MCV SIA and RI delivery, which accounted for vaccination team training and labor costs, are a simple mean of those included in the WHO-UNICEF vaccination database for comprehensive multiyear plans.^24^ The cost per participant for a measles serological survey was obtained from a study in southern Zambia.^9^

To assess differential levels of risk among districts classified as having medium outbreak risk by MRAT, we ranked these districts using MCV1 coverage estimates from the 2018 Zambia DHS. Because true district-level seroprevalence was unknown, we used modeled estimates using specimens collected from the 2016 ZamPHIA study.^25^ Input parameters and their sources are listed in Table 1. Of note, we excluded five districts lacking DHS MCV1 coverage estimates or modeled seroprevalence estimates, such that a total of 110 districts remained in the model.

**Table 1:** Deterministic model data

Variable	Value	Source
Costs (USD)		
MCV delivery cost per dose	0.97	cMYP Zambia 2010^24^
MCV materials cost per dose	0.32	Portnoy et al. 2015^23^
Community-based serological survey per participant	104	Carcelen et al. 2020^9^
Residual-based serological survey per participant	47.10	Carcelen et al. 2020^9^
Rapid diagnostic test per participant	90.83	Carcelen et al. 2020^9^
Coverage		
SIA coverage by district	0.856–0.989	Post-SIA evaluation Zambia 2016*
Proportion zero-dose among SIA-vaccinated children	0.04–0.15	Post-SIA evaluation Zambia 2016*
District routine vaccination coverage	0.675–0.983	DHS Zambia 2018^20^
District seroprevalence estimates	0.627–0.868	Carcelen et al. 2022^25^
Demographic information		
Under 5-year-old population per district	1,110–446,923	Imputed from Zambia Ministry of Health Data 2017–2019

cMYP = comprehensive multi-year strategic plans for immunization; DHS = demographic health survey; SIA = supplementary immunization activity. All prices are adjusted to June 2020 USD. *Unpublished data.

### Serological survey sample size parameters.

Serological survey sample size calculations were based on the WHO’s reference manual for vaccination cluster surveys.^26^ We assumed an α of 0.05, a power level of 0.8, and a design effect of 1.5. Owing to high fertility rates in Zambia, we expected to find a child under 5 years of age in every household. Because seroprevalence is measured at the district level, the number of strata was 1. A nonresponse rate of 36% was obtained from a serosurvey in southern Zambia, and this rate was applied to all districts.^9^ We specified our sample size calculations to detect measles immunity of 90% with a delta level of 10%. Although WHO/UNICEF Estimates of National Immunization Coverage estimate of Zambia’s 2019 nationwide MCV1 coverage was 93%,^27^ our modeled seroprevalence estimates suggest that the proportion fully vaccinated against measles was below 90% in nearly all districts. Our parameters resulted in a sample size of 119 survey participants for each district serosurvey.

### Deterministic model.

The three SIA scenarios are presented in mathematical notation in Supplement B. The outcomes of interest are the total cost of a nationwide measles SIA in Zambia for each scenario and the associated cost of vaccinating one measles zero-dose child using each method. Although we thought subnational SIAs would be more effective than RI in reaching zero-dose children, we took a conservative estimate with the base model by assuming that an invested RI system was just as effective as a subnational SIA in reaching a zero-dose child. We also included a sensitivity analysis where RI was 0.75 times (low estimate) or 1.25 times (high estimate) as effective in vaccinating zero-dose children as a subnational SIA.

The first vaccination scenario represents a standard national SIA program. All children younger than 5 years were targeted for measles vaccination in all 110 Zambian districts regardless of prior vaccination status.

Scenario 2 used a targeted approach to choose districts for a subnational measles SIA. Each of the 110 districts was assigned an outbreak risk score based on input of district-specific parameters. Risk score thresholds indicate whether a district was at low, medium, high, or very high risk of a measles outbreak in 2020 (Supplemental Table 3). Only districts classified as high or very high risk conducted a district-level SIA.

Scenario 3 was the most complex model and the only scenario to include measles serological surveys and seroprevalence estimates. Scenario 3 relied on the MRAT to classify district risk for measles outbreaks in addition to conducting measles serosurveys in medium-risk districts. High-risk and very high–risk districts administered a district-level measles SIA. Low-risk districts continued to improve upon RI to reach zero-dose children. Medium-risk districts were assessed in a multistage decision process to determine whether they received a district-level SIA, continued RI, or required the use of a measles serosurvey to assess SIA eligibility.

## STATISTICAL ANALYSES

We produced additional sensitivity analyses to account for variation among key parameters for S3a. A one-way sensitivity analysis was used to explore which model inputs had the greatest influence on SIA price (see parameters in Supplemental Table 2). The comprehensive multi-year strategic plans for immunization data used to calculate the delivery cost per dose estimate contained fewer than 20 observations (none of which included estimates specifically for Zambia), and the values were subject to wide variation, which can result in large SDs. To reduce the variation in delivery cost per dose, we opted for a 95% CI with bounds ±15% from the base value. District-specific SIA coverage estimates, DHS routine MCV1 vaccination coverage, and seroprevalence estimates each had 95% CIs. The proportion of zero-dose children reached by an SIA only exists as a point estimate, so we assumed bounds of ±15%. Since we had no variation estimates for the under 5-year-old population for each district, we assumed their 95% CI bounds to be ±15% of base values. The routine effectiveness multiplier relative to subnational SIA had a low estimate of 0.85 and a high estimate of 1.15. Base values and ranges for varied parameters can be found in Supplemental Table 6.

After the one-way sensitivity analysis was a probabilistic sensitivity analysis (PSA) to account for simultaneous variation among key input parameters. Assuming base values as medians, the R package “rriskDistributions” estimates parameter means and SDs from base values and 95% CIs. For random draws of our varying parameters, the following probabilistic distribution patterns were applied: costing parameters followed a gamma distribution, coverage parameters followed a beta distribution, and population and effectiveness parameters followed a Gaussian distribution. Per standard procedure, we ran the PSA for 10,000 cycles.

## RESULTS

### Deterministic model.

Results using the base routine effectiveness multiplier are shown in Table 2, whereas those for the low and high multipliers can be found in Supplemental Tables 4 and 5 of Supplement C, respectively. Scenario 1 yielded a total SIA cost of approximately $3.5 million, which is about $1.5 million more than the other two scenarios. Among the two tailored SIA strategies, S3 accrued a greater total cost than S2, although much of this increase was due to the addition of serological surveys. Of all scenarios, S1 vaccinated the most children as expected, although not necessarily the most zero-dose children. Assuming the base and high routine effectiveness multipliers, S2 and all S3 sub-scenarios vaccinated more zero-dose children. However, applying the low routine multiplier estimate resulted in S1 reaching the most zero-dose individuals.

**Table 2:** Results of deterministic costing model for all vaccination scenarios, assuming that investments in routine immunization are as effective as selective SIAs in reaching zero-dose children

Variable	S1	S2	S3a	S3b	S3c
Total cost (USD)	$3,446,930	$1,873,751	$2,041,889	$2,000,096	$2,030,918
Cost to immunize one child (USD)	$1.29	$1.47	$1.51	$1.48	$1.50
Total children receiving MCV	2,672,039	1,272,549	1,350,396	1,350,396	1,350,396
Cost to immunize one zero-dose child (USD)	$13.75	$7.20	$7.88	$7.72	$7.84
Total zero-dose children receiving MCV	250,776	260,145	259,076	259,076	259,076
Number of districts receiving serological survey	–	–	7	8	7
Number of medium-risk districts receiving SIA	–	–	5	5	5

MCV = measles-containing vaccine; S = scenario; SIA = supplementary immunization activity.

Of the targeted approaches, more children were vaccinated in S3 than in S2 because of the SIAs implemented in some medium-risk districts in addition to all higher risk districts, although the numbers of zero-dose children reached were similar. In the S3 sub-scenarios, seven or eight medium-risk districts were deemed eligible for measles serosurveys. For these S3 sub-scenarios, five medium-risk districts were selected for SIAs. This included both medium-risk districts eligible for SIAs because of low measles seroprevalence and low population districts where the cost of an SIA would be less than that of a community-based serological survey, as in S3a.

In terms of cost efficiencies, S1 had the highest cost to vaccinate each measles zero-dose child at $13.75. Scenario 2 had the lowest cost per zero-dose child. Among the serology-based scenarios, the use of facility-based serosurveys produced the lowest vaccination cost per measles zero-dose child, whereas community-based serosurveys commanded the highest cost, though these differences were quite small as a result of the substantial SIA delivery costs relative to total serosurvey costs.

### One-way sensitivity analysis.

**Figure 2.**
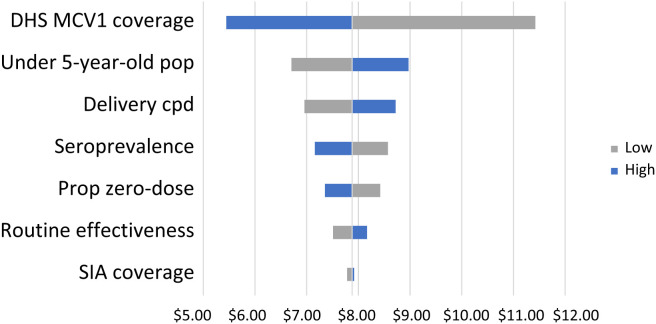
Tornado diagram assessing the impact of varied parameters on the cost to vaccinate one zero-dose child in S3a. Blue bars represent changes associated with an increase in the parameter’s value, whereas gray bars represent those with a decrease. cpd = cost per dose; DHS = demographic health survey; MCV = measles-containing vaccine; pop = population; prop = proportion; S3a = scenario 3a; SIA = supplementary immunization activity.

We ran a one-way sensitivity analysis for S3a using the upper and lower bounds of the 95% CIs for the six inputs indicated in Supplemental Table 6. A tornado diagram visualizing the variations of single-value parameter changes on the cost to immunize one measles zero-dose child is presented in Figure 2. Centering the outcome at $7.88 per zero-dose child (Table 2), DHS MCV1 RI coverage had the greatest influence on the outcome, resulting in a cost to immunize one measles zero-dose child from $5.45 to $11.42. This was followed by inputs of the target population size, MCV delivery cost per dose, seroprevalence estimate, proportion of children reached who were zero-dose, and the routine effectiveness multiplier. Costs were not significantly impacted by variation in the proportion of children under 5 years of age who were reached by our simulated SIA.

### Probabilistic sensitivity analysis.

A PSA was run for all three S3 sub-scenarios. The base PSA applied a DHS MCV1 coverage threshold of 90% and a seroprevalence threshold of 80% for ranking medium-risk districts and categorizing serological survey-eligible districts, respectively. The cost per zero-dose child was $7.83 ($6.86–$8.86) for S3a, $7.67 ($6.72–$8.68) for S3b, and $7.79 ($6.83–$8.82) for S3c.

Additional PSA runs accounted for nine different combinations of DHS MCV1 coverage (85%, 90%, or 95%) and seroprevalence values (75%, 80%, or 85%) for S3a. Controlling for DHS MCV1 coverage, changing seroprevalence thresholds appeared to have little influence on the total cost of an S3a SIA. However, when keeping the seroprevalence threshold constant, increasing the DHS MCV1 threshold increased the total SIA cost. The total cost differences between MCV1 coverage thresholds from 90% and 95% are greater than those between 85% and 90%. Detailed PSA results are shown in Supplement D.

## DISCUSSION

As districts have different measles outbreak risks, a mixed vaccination strategy may prove beneficial. Districts determined to have a high outbreak risk can conduct district-level SIAs, whereas districts with a relatively low outbreak risk can continue building upon their RI systems to overcome obstacles in reaching zero-dose children. Our analyses present an initial framework for organizing districts into risk strata to determine which type of intervention they should receive.

Our model found that targeted SIAs are more cost-efficient for reaching zero-dose children than traditional nationwide SIAs under the stated assumptions. Inclusion of serology in a tailored approach may not be prohibitively expensive, as it only slightly increases the cost of vaccinating one zero-dose child while allowing decision-makers to understand immunity profiles of selected areas. To avoid drastic cost increases, serosurveys are reserved for communities with uncertain risk classification. The published literature on targeted SIA approaches is sparse, and to our knowledge, this analysis is the first to incorporate measles serosurveys into the decision framework.

In tailoring SIAs using community-based measles serosurvey results, we found that the DHS MCV1 RI coverage threshold used to qualify medium-risk districts for serosurveys and subnational SIAs is critical for determining the cost per zero-dose child vaccinated in S3. The large range in costs appears to be due to lower-bound MCV1 coverage resulting in 28 medium-risk districts selected for subnational SIAs, whereas the upper-bound estimate qualified none of these districts for an SIA. Probabilistic sensitivity analysis results (Supplement D) also showed that adjustments to DHS MCV1 thresholds appear to impact costs substantially, indicating that there is much work to be done in exploring how inclusionary criteria affect the cost-effectiveness of targeted vaccine interventions.

Changing seroprevalence thresholds for selecting medium-risk districts for targeted SIAs did not appear to impact costs substantially. Few districts eligible for a measles serosurvey are evaluated because the cost of automatically performing a district SIA is oftentimes less than the serological survey itself. If serological surveys become less expensive to implement or are scaled to account for multiple infectious diseases, making it practical to include more districts, the seroprevalence threshold may bear greater influence in the cost of a targeted SIA.

Reaching zero-dose children specifically is a costly endeavor. A report for Gavi, the Vaccine Alliance, suggested that the cost of reaching a zero-dose child could be three to four times greater than that of reaching an already immunized child.^28^ Considering that immunizing zero-dose children is a key objective to measles control, decision-makers need to be prepared for the significant investments required to achieve measles elimination.

Our model suggests that subnational SIAs may be able to vaccinate a similar number of zero-dose children for a lower total cost than national SIAs based on our assumptions. Nationwide SIA programs are very effective in reaching zero-dose children in countries with low MCV coverage.^2^^,^^18^^,^^29^^,^^30^ However, nationwide SIAs may be less effective in countries with already high routine coverage rates, as barriers to RI for zero-dose children persist during nationwide, nonselective SIAs, resulting in revaccination of children previously vaccinated against measles.^18^^,^^31^ In addition, geospatial analyses have found variations in the spatial heterogeneity of zero-dose children throughout less-developed rural areas as well as pockets in urban centers, which puts the unvaccinated at risk of clustered outbreaks.^32^^,^^33^ This may mean that far fewer zero-dose children will be reached under S1 in practice than our model suggests and that targeted strategies are more efficient for reaching these children and reducing redundant use of resources. Furthermore, SIAs are not recommended as a permanent solution to reaching the zero-dose, as primary reliance should be focused on enhancing RI systems to better reach measles zero-dose children.^34^

Although our decision framework employed MRAT, DHS data, and serology for classifying districts into intervention categories, other methods could be considered. The MRAT was not designed to inform planning of immunization activities, and DHS data may not accurately represent communities with difficulties engaging with the health system. Novel data sources and the development of improved tools for assigning communities to interventions would be beneficial for creating an effective framework for reaching zero-dose children.

Serological surveillance has the benefit of accounting for both vaccine-induced immunity and natural immunity from prior infection.^5^ Vaccination cards, caregiver recall, and administrative data can provide unreliable estimates of vaccine coverage and population immunity,^6^^,^^35^^,^^36^ particularly in areas with weak immunization systems where zero-dose children are likely to reside. Widespread use of serological surveys is hindered by their cost, logistics, requirements of skilled personnel, assay validity, and analysis and statistical expertise.^4^ Establishing serological surveillance networks with existing government health systems and laboratories may minimize these challenges. Although material costs and per-dose vaccine costs are likely to remain firm, there may be flexibility in lowering field costs for obtaining samples, which comprise a significant proportion of the total cost of a survey. Nesting serological sampling in other surveys can ease the data collection process, which accounts for a significant proportion of the costs of serological surveillance.^9^ In the case of our model’s residual samples scenario, we did not apply serosurveys to enough districts for the cost-saving measures of nested sampling to have a substantial impact on cost. It may be possible that nesting serosurveys that use multiplex assays is a more effective cost-saving measure by expanding the scope of serosurveillance to a group of communicable diseases.

Despite requiring additional resources beyond RI costs, MCV vaccination campaigns can be cost-effective in reducing measles burden. A 2004 study in Zambia found that an MCV vaccination program utilizing both RI and SIA was more cost-effective than programs using RI alone.^37^ However, these national SIAs may no longer be effective in vaccinating zero-dose children with MCV, and therefore no longer cost-effective, in countries with high MCV coverage, as campaigns revaccinate children with MCV instead of addressing the problem of zero-dose.^38^ The cost-effectiveness of SIAs, whether national or subnational, also depends on the outbreak risk remaining after a campaign. Identifying the threshold of national versus subnational SIA cost-effectiveness is an area of future work.

Our model is hindered by the lack of empirical data on the additional costs and benefits of conducting a targeted SIA versus a nationwide SIA, requiring assumptions for these inputs. Future analyses may explore the impact of economies of scale on serological survey cost per participant. Using DHS MCV1 coverage to rank medium-risk districts by relative risk priority does not account for immunity acquired via prior infection, so there is a need to explore better methods, such as serology, for targeting high outbreak risk areas for intervention.^39^ Because age-specific population estimates were unavailable for most districts, we had to extrapolate the under 5-year-old population. In S2 and S3, we made assumptions on the effectiveness of additional routine system investments to immunize zero-dose children. These assumptions may not allow us to get an accurate cost under a real-world scenario; however, sensitivity analyses indicate that routine effectiveness is not a key factor in program costs.

Another neglected factor is time. Planning, implementing, and analyzing the results of serological surveys could further contribute to the delay of a vaccination program. The model may need a quantifiable measure of how the delays associated with acquiring serological data ultimately impact the utility of using such an approach.

Vaccination of zero-dose children poses a significant hurdle in the last steps toward measles elimination in areas with high MCV coverage. We present an initial framework to conceptualize the classification of districts into priority groups while exploring the possible use of measles serosurveys to assess community immunity to help inform decision-making. For a model like this to work, high-quality vaccination coverage, surveillance, and demographic data need to be available at the subnational level for accurate outbreak risk classification. Future research may propose further developments in the risk categorization framework for targeted vaccination campaigns and assess or improve the cost feasibility of employing technologies for locating those who are zero-dose.

## Supplemental Materials

10.4269/ajtmh.23-0412 Supplemental Materials
