# Supplementary material for: Modeling the Cost of Vaccinating a Measles Zero-Dose Child in Zambia Using Three Vaccination Strategies
Source: Am J Trop Med Hyg. 2024 May 21;111(1):121–8. doi: 10.4269/ajtmh.23-0412 (PMC11229634; doi:10.4269/ajtmh.23-0412)
Supplement: Supplemental Materials [file tpmd230412.SD1.pdf]

Manuscript: Modeling the cost of vaccinating a measles zero-dose child in Zambia using three vaccination strategies

Supplement A: District Descriptive Statistics

If we take the estimated target populations for each district, apply the respective MCV1 coverage we would expect from a nationwide SIA, we can make a rough prediction on how zero-dose children vaccinated by SIA may be distributed among district risk types. In Table 1, we see that the overall MCV1 coverage is high among all district risk types. However, we see that 53.2% of zero-dose children reached via a nationwide SIA reside in the high risk districts. This is likely because high risk districts on average contain a high proportion of children in our target age range. It is also worth noting that low risk and very high risk districts appear to contain a relatively low proportion of these zero-dose individuals, suggesting that SIA efforts targeting the other two district types may be more effective in reaching greater quantities of zero-dose children.

Supplementary Table 1. District risk type aggregate characteristics. All calculations assume a standard nationwide SIA.

	Low risk	Medium risk	High risk	Very high risk
**Average MCV1 coverage**	96.0%	94.8%	95.2%	92.3%
**Allocation of zero-dose children among risk groups**	8.4%	32.6%	53.2%	5.8%
**Average under 5 population per district**	18,158	20,872	41,024	13,871
**Total number of districts**	29	49	25	7
**Proportion of children vaccinated who are zero-dose**	4%	8%	13%	15%

Manuscript: Modeling the cost of vaccinating a measles zero-dose child in Zambia using three vaccination strategies

Supplementary Table 2. Model assumptions

**Proportion of children reached who are zero dose**
• Low risk: 4%
• Medium risk: 8%
• High risk: 13%
• Very high risk: 15%
**Additional children vaccinated via selective SIA**
• Scenario 1: Reference
• Scenario 2: 10%
• Scenario 3: 5%
**Districts not selected for district-level SIA do not contribute to costs or effects**
**Economies of scale are not considered for costs**

Supplementary Table 3. MRAT risk score breakdown.

Risk score (RS)	Risk classification	Number of districts
RS < 48	Low risk	29
48 ≤ RS < 55	Medium risk	52
55 ≤ RS < 61	High risk	25
61 ≤ RS	Very high risk	8

Supplement B: Methods and Equations

Threshold populations for deciding that automatically implementing an SIA is more cost-effective than conducting a serosurvey:

The threshold population sizes for deciding whether a district should be selected for a serological survey determine was determined by multiplying 119, the sample size per district needed to detect a seroprevalence rate of 90% with a delta value of 10, by the cost of a serological survey using one of three methods: community-based serological survey, residual-based serological survey, and IgG point-of-contact diagnostic tests. The product is then divided by the cost to vaccinate a child against measles to determine how many children could have otherwise been

vaccinated for the price of a district-level serological survey. The threshold population size is 9,594 using a community-based serological survey, 4,345 for a residual-based serological survey, and 8,379 for an IgG point-of-contact diagnostic tests.

S1: Non-specific nationwide SIA

$$SIA\_Total_1 = \sum_{i=1}^{110} x_{1,i} * x_{2,i} * x_3 \quad (1)$$

$$ZD\_Cost_1 = \frac{SIA\_Total_1}{\sum_{i=1}^{110} x_{1,i} * x_{2,i} * x_4} \quad (2)$$

Variable list:

- *SIA_Total₁*: the total cost of an SIA for S1
- *ZD_Cost₁*: the cost to vaccinate one zero-dose child for S1
- $x_{1,i}$: the total population under 5 years of age for district *i*
- $x_{2,i}$: the proportion of children under 5 years of age in district *i* who actually receive a dose of MCV during SIA
- x_3: the sum of the vaccine materials cost per dose and the SIA vaccine delivery cost per dose
- x_4: the proportion of children vaccinated by SIA who are zero-dose.

S2: Using MRAT to target districts for SIA

$$SIA\_Total_2 = \sum_{i=1}^{110} x_{1,i} * x_{2,i} * (x_3 * HR_i + x_4 * (1 - HR_i)) \quad (3)$$

$$ZD\_Cost_2 = \frac{SIA\_Total_2}{\sum_{i=1}^{110} x_{1,i} * x_{2,i} * x_5 * HR_i} \quad (4)$$

Variable list:

- *SIA_Total₂*: the total cost of an SIA for S2
- *ZD_Cost₂*: the cost to vaccinate one zero-dose child for S2

- $x_{1,i}$: the total population under 5 years of age for district *i*
- $x_{2,i}$: the proportion of children under 5 years of age in district *i* who actually receive a dose of MCV during SIA
- x_3: the sum of the vaccine materials cost per dose and the SIA vaccine delivery cost per dose
- x_4: the sum of the vaccine materials cost per dose and the *routine* vaccine delivery cost per dose per person
- HR_i: equals 1 when MRAT considers district *i* to have high or very high outbreak risk and equals 0 when otherwise. This indictor will therefore allows for different costs and numbers of zero-dose children vaccinated depending on whether a district receives a subnational SIA or RI investment.

S3: Using MRAT to target districts for SIA and serological survey

$$SIA\_Total_3 = \sum_{i=1}^{110}[x_{1,i} * x_{2,i} * x_3 * HR_i + x_{1,i} * x_{2,i} * x_3 * MR_{DHS_i} * (1 - survey_i) + (x_{1,i} * x_{2,i} * x_3 + sero\_cost_i * survey_i) * MR_{DHS_i} * sero_i + sero\_cost_i * sero_i * MR_{DHS_i} * (1 - sero_i)] \quad (5)$$

$$ZD\_Cost_3 = \frac{SIA\_Total_3}{\sum_{i=1}^{110}[x_{1,i}*x_{2,i}*x_5*HR_i+x_{1,i}*x_{2,i}*MR_{DHS_i}*(1-survey_i)+x_{1,i}*x_{2,i}*x_3*MR_{DHS_i}*sero_i]} \quad (6)$$

Variable list:

- SIA_Total_3: the total cost of an SIA for S3
- ZD_Cost_3: the cost to vaccinate one zero-dose child for S3
- $x_{1,i}$: the total population under 5 years of age for district *i*

- $x_{2,i}$: the proportion of children under 5 years of age in district *i* who actually receive a dose of MCV during SIA
- x_3: the sum of the vaccine materials cost per dose and the SIA vaccine delivery cost per dose
- x_4: the sum of the vaccine materials cost per dose and the *routine* vaccine delivery cost per dose per person
- HR_i: equals 1 when MRAT considers district *i* to have high or very high outbreak risk and equals 0 when otherwise. This indictor will therefore allows for different costs and numbers of zero-dose children vaccinated depending on whether a district receives a subnational SIA or RI investment.
- MR_DHS_i is an indicator for medium risk districts that qualify for serological survey consideration based on DHS reported MCV1 coverage – for the deterministic model, this variable is assigned a value of 1 if the MCV1 coverage rate of the medium risk district is less than 90%.
- $survey_i$ indicates whether the cost of performing a serological survey is less than that of performing an SIA for district *i*
- $sero_i$: an indicator for whether the underlying seroprevalence of district *i* confirms that an SIA should be performed – in the deterministic model, this variable is assigned a value of 1 if the modeled seroprevalence of the medium risk district under serological survey consideration is less than 80%.

- *sero_cost$_i$*: the total cost of assessing serology in 119 individuals in a district – this value is: $12,376 with community-based serology, $5,605 with facility-based serology, and $10,809 with rapid diagnostic testing.

Supplement C: Additional Deterministic Results

Supplementary Table 4. Results of deterministic costing model for all vaccination scenarios, assuming that investments in routine immunization are 25% less effective than selective SIAs in reaching zero-dose children.

	S1	S2	S3a	S3b	S3c
Total cost (USD)	$3,446,930	$1,781,365	$1,956,660	$1,914,867	$1,945,689
Cost to immunize one child (USD)	$1.29	$1.43	$1.48	$1.44	$1.47
Total children receiving MCV	2,672,039	1,245,925	1,325,654	1,325,654	1,325,654
Cost to immunize one zero-dose child (USD)	$13.75	$7.63	$8.35	$8.17	$8.30
Total zero-dose children receiving MCV	250,776	233,521	234,334	234,334	234,334
Number of districts receiving serological survey	--	--	7	8	7
Number of medium-risk districts receiving SIA	--	--	5	5	5

Supplementary Table 5. Results of deterministic costing model for all vaccination scenarios, assuming that investments in routine immunization are 25% more effective than selective SIAs in reaching zero-dose children.

	S1	S2	S3a	S3b	S3c
Total cost (USD)	$3,446,930	$1,966,136	$2,127,118	$2,085,325	$2,116,148
Cost to immunize one child (USD)	$1.29	$1.51	$1.55	$1.52	$1.54
Total children receiving MCV	2,672,039	1,299,173	1,375,139	1,375,139	1,375,139
Cost to immunize one zero-dose child (USD)	$13.75	$6.86	$7.49	$7.35	$7.46
Total zero-dose children receiving MCV	250,776	286,769	283,819	283,819	283,819
Number of districts receiving serological survey	--	--	7	8	7
Number of medium-risk districts receiving SIA	--	--	5	5	5

Manuscript: Modeling the cost of vaccinating a measles zero-dose child in Zambia using three vaccination strategies

Supplement D: Probabilistic Sensitivity Analysis

Supplementary Table 6. Sensitivity analysis input data. All prices are adjusted to June 2020 USD.

	Base Value	95% CI	Distribution Pattern
**Costing**			
MCV delivery cost per dose (USD)	0.97	0.83-1.12	--
MCV materials cost per dose (USD)*	0.32	--	--
Community-based serological survey per participant (USD)*	104	--	--
Residual-based serological survey per participant (USD)*	47.10	--	--
Rapid diagnostic test per participant*	90.83	--	--
**Coverage**			
SIA coverage by district (%)	85.6-98.9	Varies	Beta
Effectiveness multiplier in reaching zero-dose for districts improving routine immunization systems rather than implementation of SIAs	1.0	0.75-1.25	Gaussian
Proportion zero-dose among SIA-vaccinated children (%)	14	9-19	Gaussian
DHS district MCV1 vaccination coverage (%)	67.5-98.3	Varies	Beta
District seroprevalence estimates (%)	62.7-86.8	Varies	Beta
**Demographic information**			
Under 5 yrs population per district	1,110-446,923	Varies	Gaussian

* Indicates parameter was not varied during sensitivity analysis.

The base case PSA assumes the decision threshold parameters used in the deterministic model for all S3 sub-scenarios: the DHS MCV1 RI coverage is set at 90% and the seroprevalence estimate is set at 80% (Supplementary Table 5). After setting a seed in the statistical software, the base case PSA was run for 10,000 iterations. Using a dataset with the results from each of the 10,000 runs of the model, we extract the median and middle 95% range of values for each outcome of interest. The median SIA total cost is $2,028,521 (95% CI $1,775,167-2,299,950) for S3a, $1,987,270 (95% CI $1,738,168-2,250,851) for S3b, and $2,018,871 (95% CI $1,766,864-2,288,097) for S3c. Values for the cost to immunize one zero-dose child are as follows: S3a is $7.83 (95% CI $6.86-8.86), S3b is $7.67 (95% CI $6.72-8.68), and S3c is $7.79 (95% CI $6.83-8.82).

Manuscript: Modeling the cost of vaccinating a measles zero-dose child in Zambia using three vaccination strategies

Given that all S3 sub-scenarios PSAs are run on the same seed, they all share the same values for zero-dose children receiving MCV, districts receiving serological survey, and medium risk districts receiving SIA.

Supplementary Table 7. Results of probabilistic sensitivity analysis for S3 using base case decision thresholds of 90% DHS MCV1 coverage and 80% seroprevalence estimates.

**All S3 (Note: these values are the same for all S3 sub-scenarios)**	Median	95% Range
Total zero-dose children receiving MCV	259,157	245,182 – 273,589
Number of districts receiving serological survey	6	4 – 9
Number of medium risk districts receiving SIA	6	3 – 10
**S3a only**	Median	95% Range
Total SIA cost (USD)	$2,028,521	$1,775,167 – 2,299,950
Cost to immunize one zero-dose child (USD)	$7.83	$6.86 – 8.86
**S3b only**	Median	95% Range
Total SIA cost (USD)	$1,987,270	$1,738,168 – 2,250,851
Cost to immunize one zero-dose child (USD)	$7.67	$6.72 – 8.68
**S3c only**	Median	95% Range
Total SIA cost (USD)	$2,018,871	$1,766,864 – 2,288,097
Cost to immunize one zero-dose child (USD)	$7.79	$6.83 – 8.82

Using S3a, run nine PSAs with different parameterizations of the DHS MCV1 coverage rate, for qualifying medium risk districts for serological survey, and of the seroprevalence threshold, for determining whether a serology-qualifying district should receive a serological survey or an automatic SIA. In each PSA, we designate a DHS MCV1 coverage threshold of 85%, 90%, or 95% and a seroprevalence estimate threshold of 75%, 80%, or 85%.

Keeping DHS MCV1 RI coverage rates consistent, seroprevalence thresholds appear to have little influence on the total cost of an S3a SIA (Supplementary Table 6). However, when conditioning on a single seroprevalence estimate, there is a positive trend between DHS MCV1 RI coverage thresholds and total SIA cost. Of note, when controlling for the seroprevalence threshold, cost differences between MCV1 coverage thresholds from 90% and 95% are greater

than that between 85% and 90%. Supplementary Table 7 repeats these findings but changes the output of interest to the cost of vaccinating one zero-dose child.

Supplementary Table 8. Median values of total SIA costs for S3a probabilistic sensitivity analyses with varied DHS MCV1 coverage and seroprevalence thresholds. All costs are reported in USD; 95% confidence intervals are given in paratheses.

	DHS MCV1 Coverage		
	85%	90%	95%
**Seroprevalence**			
75%	$1,911,563<br>($1,680,632 – 2,152,743)	$1,916,796<br>($1,685,112 – 2,158,854)	$ 1,950,995<br>($1,710,207 – 2,202,319)
80%	$2,010,663<br>($1,762,386 – 2,266,711)	$2,028,521<br>($1,775,167 – 2,299,950)	$2,112,597<br>($1,836,175 – 2,416,914)
85%	$2,358,205<br>($2,076,164 – 2,646,486)	$2,458,499<br>($2,148,803 – 2,788,241)	$ 2,730,280<br>($2,366,009 – 3,118,776)

Supplementary Table 9. Median values of cost to vaccinate one zero-dose child for S3a probabilistic sensitivity analyses with varied DHS MCV1 coverage and seroprevalence thresholds. All costs are reported in USD; 95% confidence intervals are given in paratheses.

	DHS MCV1 Coverage		
	85%	90%	95%
**Seroprevalence**			
75%	$7.38<br>($6.49 – 8.28)	$7.40<br>($6.51 – 8.30)	$7.53<br>($6.61 – 8.48)
80%	$7.75<br>($6.82 – 8.73)	$7.83<br>($6.86 – 8.86)	$8.16<br>($7.10 – 9.30)
85%	$9.10<br>($8.02 – 10.22)	$9.49<br>($8.29 – 10.77)	$10.56<br>($9.14 – 12.06)
